# Supplementary material for: Ablation of TrkB from Enkephalinergic Precursor-Derived Cerebellar Granule Cells Generates Ataxia
Source: Biology (Basel). 2024 Aug 20;13(8):637. doi: 10.3390/biology13080637 (PMC11351323; doi:10.3390/biology13080637)
Supplement: Supplementary file 1 [file biology-13-00637-s001.zip › Supplementary materials.pdf]

Supplementary Materials

Ablation of TrkB from enkephalinergic precursor-derived cerebellar granule cells generates ataxia.

Elena Eliseeva [1], Mohd Yaseen Malik [1] and Liliana Minichiello [1,*]

Supplementary Table S1. Antibodies used in immunofluorescence experiments.

**Name**	**Type**	**Source**	**Identifier**	**Dilution**
mouse anti-NeuN	Primary	Millipore	MAB377	1:500
rabbit anti-PENK	Primary	Neuromics	Ra-14124	1:1000
mouse anti-calbindin D-28k	Primary	Swant	300	1:5000
goat anti-mouse Alexa Fluor 488	Secondary	Jackson	A-11029	1:1000
goat anti-rabbit Alexa Fluor 488	Secondary	Jackson	A-11008	1:1000

Supplementary Table S2. Antibodies used in immunoblotting experiments.

Name	Type	Source	Identifier	Dilution	Gel %	Protein (ug)
rabbit anti-TrkB	Primary	Cell signaling	4603	1:250	10%	40
rabbit recombinant monoclonal anti-synaptophysin	Primary	Abcam	ab52636	1:1000	12%	30
mouse anti-calbindin D-28k	Primary	Swant	300	1:500	12%	40
mouse anti-GAD67	Primary	Chemicon	MAB5406	1:5000	10%	40
rabbit anti-PSD95	Primary	Cell signaling	3450	1:1000	10%	30
mouse anti-gamma Tubulin	Primary	abcam	ab11316	1:5000	10-12%	30-40
goat anti-rabbit IRDye 680RD	Secondary	LI-COR	926-68071	1:20,000	10-12%	30-40
goat anti-mouse IRDye 800CW	Secondary	LI-COR	926-32210	1:20,000	10-12%	30-40

Supplementary Table S3. The CatWalk XT parameters included in this study. The parameters were grouped into three categories: kinetic, temporal, and interlimb coordination [1,2]. Definitions of the parameters are those listed in the CatWalk XT (version 10.6) Reference Manual. RF, right forepaw; RH, right hindpaw; LF, left forepaw; LH, left hindpaw

Group	Parameter	Definition
Temporal	Stand (s) mean	Duration of contact of a paw with the glass plate.
Temporal	Swing (s) mean	Duration of no contact of a paw with the glass plate.
Temporal	Duty cycle (%) mean	Stand as a percentage of Step Cycle (the latter is the time in seconds between two consecutive initial contacts of the same paw).
Temporal	Step cycle (s) mean	Time between two consecutive initial contacts of the same paw.
Kinetic	Other statistics average speed	Average speed of the selected steps in the data selection.
Kinetic	Other statistics maximum variation (%)	Maximum variation in average speed of the selected steps in the data section.
Kinetic	Other statistics cadence (steps/s)	Frequency of the selected steps in the data selection, expressed in steps per second.
Kinetic	Swing speed (cm/s) mean	Speed of the paw during Swing.
Kinetic	Stand index	A measure for the speed at which the paw loses contact with the glass plate.
Interlimb coordination	Base of support (cm) mean	Average width between either the front paws or the hind paws.
Interlimb coordination	Stride length (cm) mean	Distance between successive placements of the same paw.
Interlimb coordination	Print positions	Distance between the position of the hind paw and the position of the previously placed front paw on the same side of the body and in the same Step Cycle.
Interlimb coordination	Step sequence CA (%)	The percentage of the total number of the cruciate footfall pattern sequence of RF-LF-RH-LH
Interlimb coordination	Step sequence CB (%)	The percentage of the total number of the cruciate footfall pattern sequence of LF-RF-LH-RH
Interlimb coordination	Step sequence AA (%)	The percentage of the total number of the alternate footfall pattern sequence of RF-RH-LF-LH
Interlimb coordination	Step sequence AB (%)	The percentage of the total number of the alternate footfall pattern sequence of LF-RH-RF-LH
Interlimb coordination	Step sequence RA (%)	The percentage of the total number of the rotary footfall pattern sequence of RF-LF-LH-RH
Interlimb coordination	Step sequence RB (%)	The percentage of the total number of the rotary footfall pattern sequence of LF-RF-RH-LH
Interlimb coordination	Step sequence regularity index (%)	Number of normal step sequence patterns relative to the total number of paw placements.
Interlimb coordination	Couplings	Temporal relationship between placements of two paws within a Step Cycle. In this parameter, a target paw can never precede an anchor paw. The value of Couplings ranges between 0 and 100%.

BAC-*Penk-Cre*$^{tg/+}$; Ai9

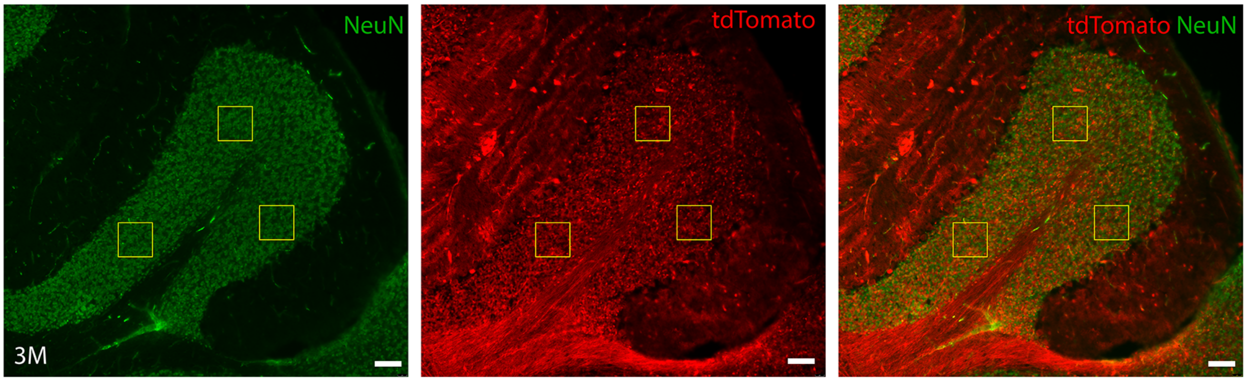

Supplementary Figure S1. Illustration of the analysis to estimate the proportion of granule cells that underwent recombination (pyramis pictured). Three regions of interest (ROI) of 10,000µm^2 are randomly chosen in the granule cell layer of three selected lobules per section. NeuN-positive (green channel) cells, labelling granule cells, are first counted in each ROI, after which those NeuN-positive cells that are also tdTomato-positive are counted in the red channel. 6 sections were analysed, three from sagittal level 11 and three from sagittal level 13, all from the same 3M *BAC-Penk-Cretg/+;Ai9* mouse. Scale bar = 75µm.

BAC-*Penk-Cre*$^{tg/+}$; Ai9

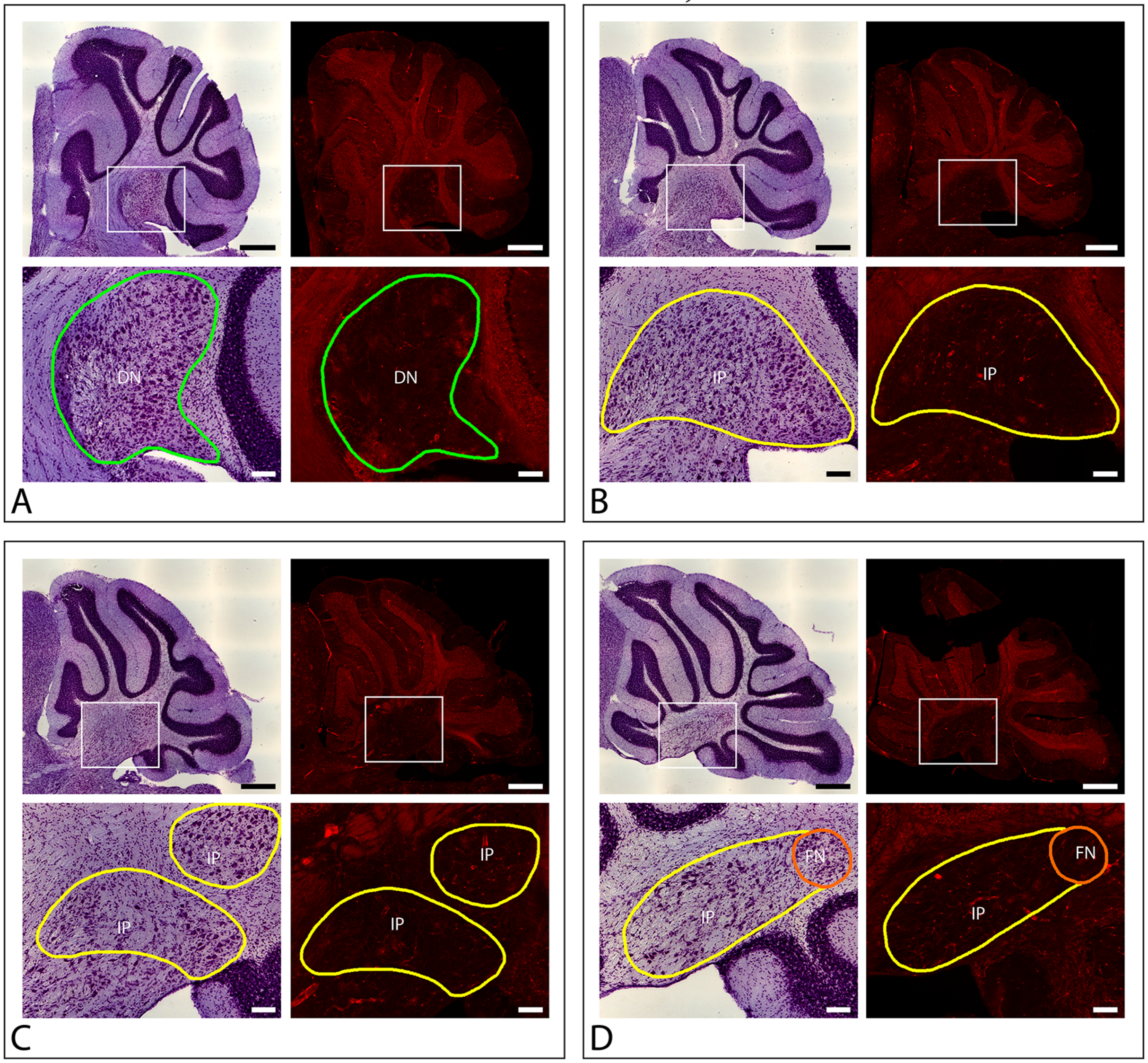

Supplementary Figure S2. Recombination in the cerebellar nuclei of 3M BAC-*Penk-Cre*$^{tg/+}$;Ai9 mice. (A-D) Representative cresyl violet stained sagittal cerebellar sections and adjacent immunofluorescent cerebellar sections from a 3M BAC-*Penk-Cre*$^{tg/+}$;Ai9 mouse demonstrate that Cre-mediated tdTomato expression occurred in only a handful of cells in the dentate (A), interposed (B, C, D), and fastigial (D) nuclei. Scale bars: 500μm in (A-D) and 100 μm in the respective insets. DN – dentate nucleus, IP – interposed nucleus, FN – fastigial nucleus.

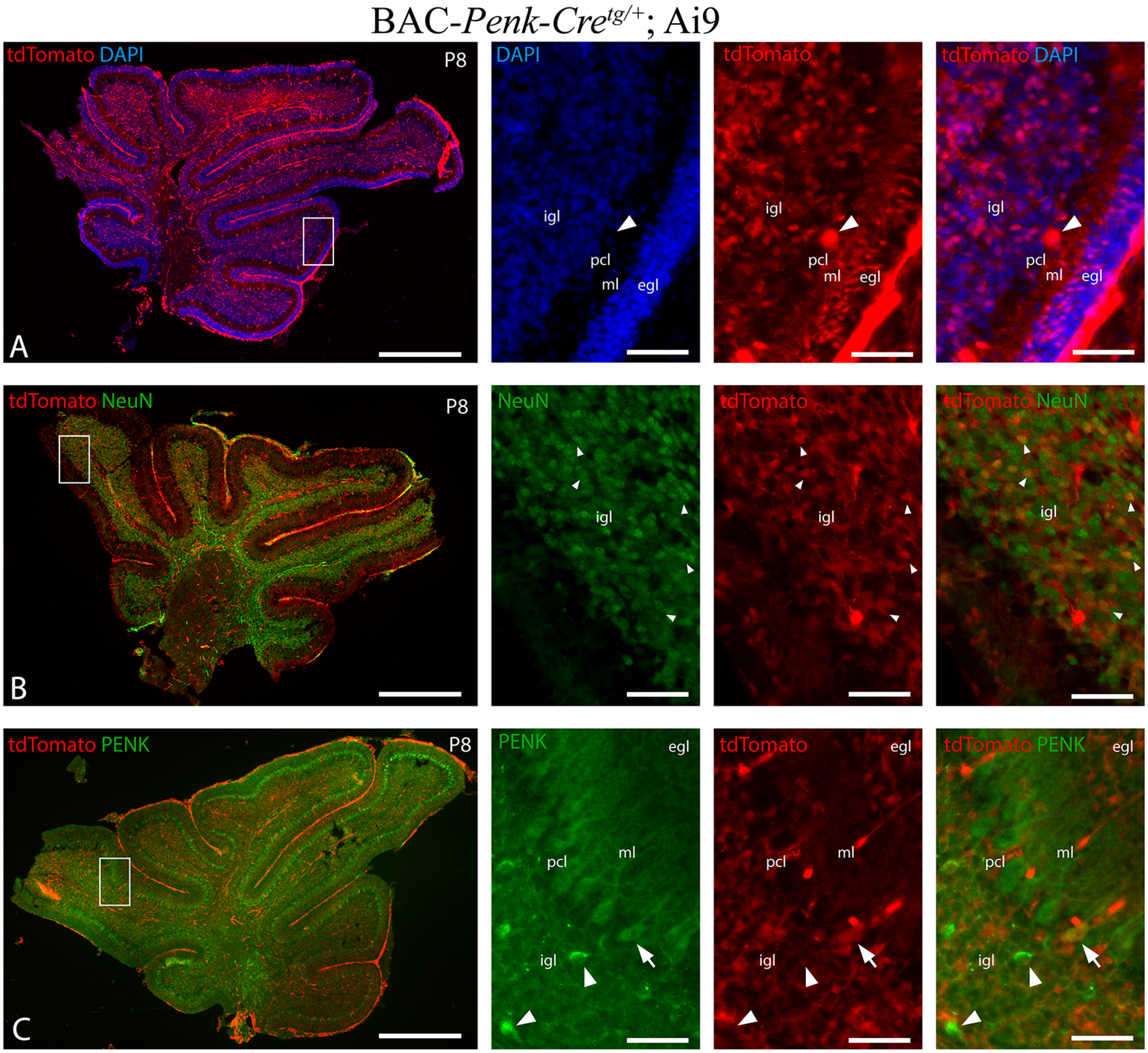

Supplementary Figure S3. Only a subset of GCs express tdTomato at P8, while Penk expression is absent at this stage. (A) DAPI-stained (blue) representative sagittal section from the cerebellum of P8 BAC-*Penk-Cre*$^{tg/+}$;Ai9 mouse reveals that Cre-mediated tdTomato expression occurred in the external and internal granule cell layers, as well as in some Purkinje cells. The arrowhead indicates a Purkinje cell. (B) NeuN immunostaining (green) on a sagittal cerebellar section of P8 BAC-*Penk-Cre*$^{tg/+}$;Ai9 mouse demonstrates that only a subset of GCs (yellow) in the internal granule cell layer undergoes Cre-recombination. Arrowheads indicate GCs positive for tdTomato. (C) PENK immunostaining (green) on a sagittal cerebellar section of P8 BAC-*Penk-Cre*$^{tg/+}$;Ai9 mouse reveals that Golgi cells and Purkinje cells are enkephalinergic at this stage, whereas GCs are not. Arrowheads and the arrow indicate enkephalinergic tdTomato-negative Golgi cells and the enkephalinergic tdTomato-positive Purkinje cell. Scale bars: 500μm in (A-C) and 50μm in respective insets. igl – internal granular layer, pcl – Purkinje cell layer, ml – molecular layer, egl – external granular layer.

BAC-*Penk-Cre*$^{tg/+}$; Ai9

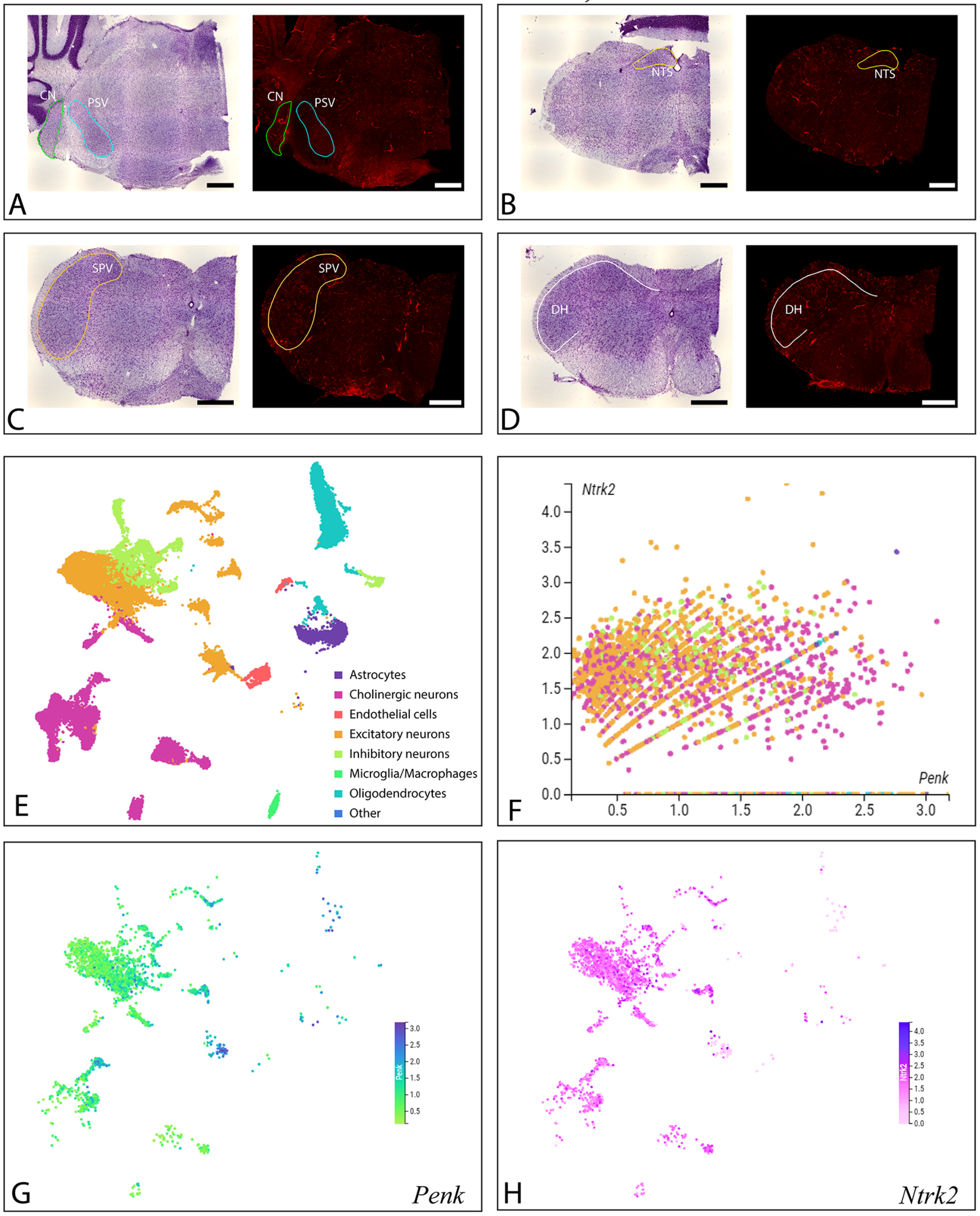

Supplementary Figure S4. Recombination in the brainstem and spinal cord of 3M BAC-*Penk-Cre*$^{tg/+}$;Ai9 mice. (A-D) Representative cresyl violet stained coronal brainstem (A-C) and spinal cord (D) sections and adjacent immunofluorescent brainstem sections from a 3M BAC-*Penk-Cre*$^{tg/+}$;Ai9 mouse demonstrate that scattered Cre-mediated tdTomato expression could be observed in cochlear nucleus complex and principal sensory nucleus of the trigeminal nerve (A), nucleus of the solitary tract (B), spinal nucleus of the trigeminal nerve (C) and in the dorsal horn of the spinal cord (D). (E-H) UMAP visualisation (E, G, H) and plot of *Penk* expression against *Ntrk2* expression (F) based on the publicly available snRNA-Seq dataset [3,4] from adult (P100-150) mouse spinal cord (cervical, thoracic and lumbar regions combined), coloured by cell identity (E, F), and log-normalised expression of *Penk* (G) and *Ntrk2* (H). Only a subset of cells with non-zero *Penk* expression was plotted in (F-H). It appears that co-expression of *Penk* and *Ntrk2* is low in the mouse spinal cord. Scale bars: 500μm. CN – cochlear nucleus complex, PSV – principal sensory nucleus of the trigeminal nerve, NTS – nucleus of the solitary tract, SPV – spinal nucleus of the trigeminal nerve, DH – dorsal horn

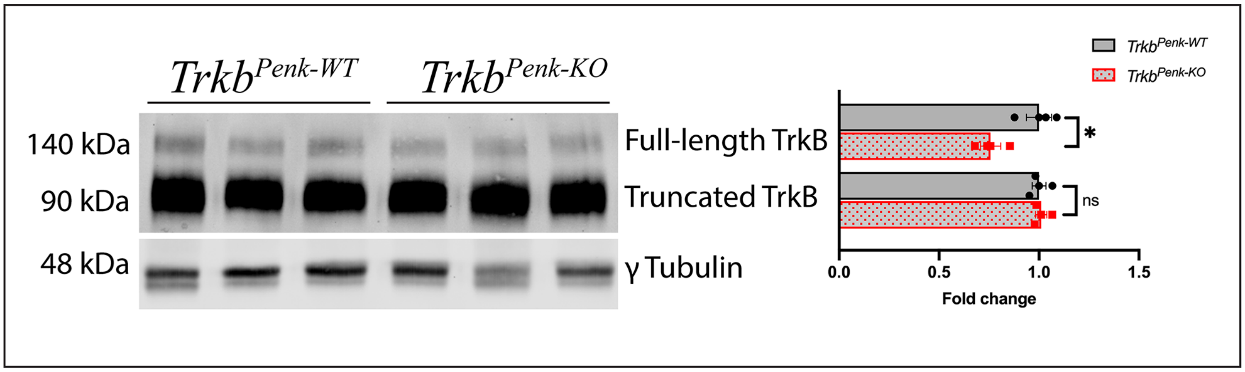

Supplementary Figure S5. Decrease in full-length TrkB in the cerebellum of 3M *Trkb*$^{\mathit{Penk\text{-}KO}}$ mice. Western blot analysis of cerebellar lysates from 3M *Trkb*$^{\mathit{Penk\text{-}WT}}$ and *Trkb*$^{\mathit{Penk\text{-}KO}}$ mice showed a significant reduction in full-length TrkB levels in mutants, $t(4)=2.98$, $p=0.041$. Truncated TrkB levels were unaffected in *Trkb*$^{\mathit{Penk\text{-}KO}}$ mice, $t(4)=0.23$, $p=0.828$. Controls, $n=3$; mutants, $n=3$; all female mice.

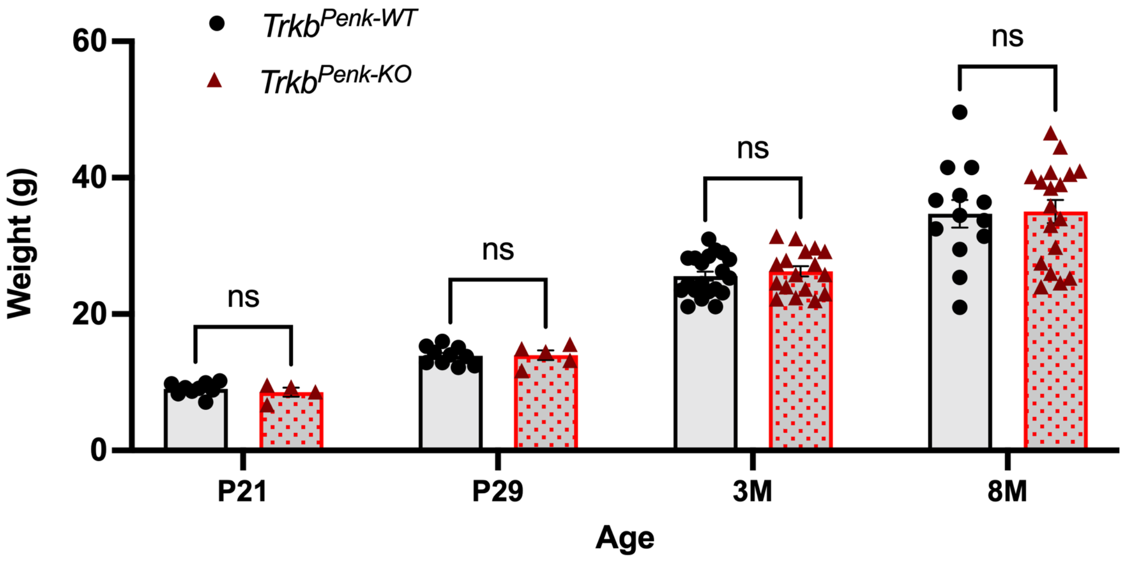

Supplementary Figure S6. Body weight analysis across different age groups.
Comparison analysis revealed no differences in body weight of *Trkb*$^{Penk\text{-}KO}$mice and controls across various age groups. Data from both sexes were pooled together for analysis as there was no interaction between sex and genotype for any age group analysed, (P21, n = 9 controls (3 males and 6 females), and 4 mutants (3 males and 1 female), $t(11)$=0.745, p=0.472; P29, n = 10 controls (5 males and 5 females), and 5 mutants (3 males and 2 females), $t(13)$=0.106, p=0.917; 3M, n = 20 controls (9 males and 11 females), and 17 mutants (9 males and 8 females), $t(35)$=0.678, p=0.503; 8M, n = 13 controls (6 males and 7 females), and 18 mutants (8 males and 10 females), $t(29)$=0.126, p=0.901. Values are means ± SEM. p statistic from unpaired, two-tailed, Student's t-test.

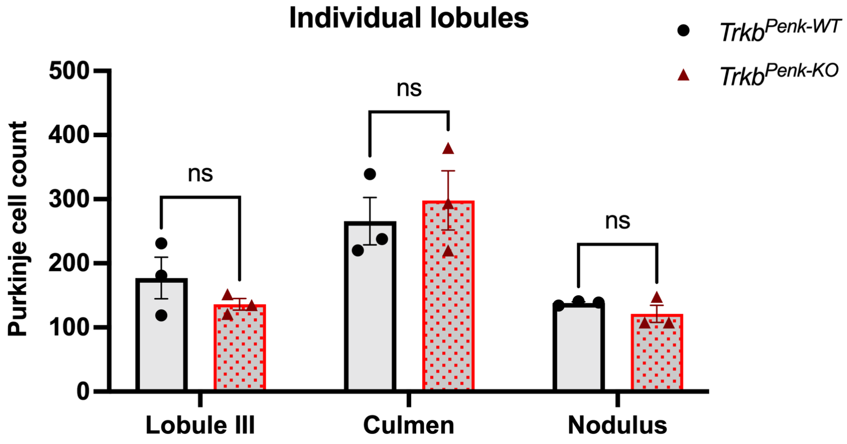

Supplementary Figure S7. Purkinje cell counts in selected lobules of *Trkb*$^{Penk\text{-}KO}$ cerebella.
A scatter bar plot showing no significant differences in Purkinje cell counts of selected individual lobules at 4M between *Trkb*$^{Penk\text{-}KO}$ mice and controls. Lobule III, $t(4)=1.220$, $p=0.290$. Culmen: $t(4)=0.546$, $p=0.614$. Nodulus: $t(4)=1.235$, $p=0.284$. Controls, $n=3$; mutants, $n=3$; all female mice. Values are means ± SEM. p statistic from unpaired, two-tailed, Student's t-test.

Supplementary Video S1. Ledge test performance of a 3-month-old control.

Supplementary Video S2. Ledge test performance of a 3-month-old mutant.

References

1. Caballero-Garrido, E.; Pena-Philippides, J.C.; Galochkina, Z.; Erhardt, E.; Roitbak, T. Characterization of Long-Term Gait Deficits in Mouse dMCAO, Using the CatWalk System. *Behavioural Brain Research* **2017**, *331*, 282–296, doi:10.1016/j.bbr.2017.05.042.
2. Timotius, I.K.; Roelofs, R.F.; Richmond-Hacham, B.; Noldus, L.P.J.J.; von Hörsten, S.; Bikovski, L. CatWalk XT Gait Parameters: A Review of Reported Parameters in Pre-Clinical Studies of Multiple Central Nervous System and Peripheral Nervous System Disease Models. *Frontiers in Behavioral Neuroscience* **2023**, *17*.
3. Blum, J.A.; Klemm, S.; Shadrach, J.L.; Guttenplan, K.A.; Nakayama, L.; Kathiria, A.; Hoang, P.T.; Gautier, O.; Kaltschmidt, J.A.; Greenleaf, W.J.; et al. Single-Cell Transcriptomic Analysis of the Adult Mouse Spinal Cord Reveals Molecular Diversity of Autonomic and Skeletal Motor Neurons. *Nat Neurosci* **2021**, *24*, 572–583, doi:10.1038/s41593-020-00795-0.
4. Alkaslasi, M.R.; Piccus, Z.E.; Hareendran, S.; Silberberg, H.; Chen, L.; Zhang, Y.; Petros, T.J.; Le Pichon, C.E. Single Nucleus RNA-Sequencing Defines Unexpected Diversity of Cholinergic Neuron Types in the Adult Mouse Spinal Cord. *Nat Commun* **2021**, *12*, 2471, doi:10.1038/s41467-021-22691-2.
